# Novel human melanoma brain metastasis models in athymic nude fox1^nu^ mice: Site‐specific metastasis patterns reflecting their clinical origin

**DOI:** 10.1002/cam4.4334

**Published:** 2021-10-06

**Authors:** Henrik A. Svendsen, Torstein R. Meling, Vigdis Nygaard, Stein Waagene, Hege Russnes, Siri Juell, Siril G. Rogne, Jens Pahnke, Eirik Helseth, Øystein Fodstad, Gunhild M. Mælandsmo

**Affiliations:** ^1^ Institute of Clinical Medicine Faculty of Medicine University of Oslo Oslo Norway; ^2^ Department of Neurosurgery Oslo University Hospital Oslo Norway; ^3^ Department of Tumor Biology Institute for Cancer Research Oslo University Hospital‐Radiumhospitalet Oslo Norway; ^4^ Department of Neurosurgery Geneva University Hospitals Geneva Switzerland; ^5^ Faculty of Medicine University of Geneva Geneva Switzerland; ^6^ Department of Pathology Oslo University Hospital Oslo Norway; ^7^ LIED University of Lübeck Jena Germany; ^8^ Department of Pharmacology Medical Faculty University of Latvia Riga Latvia; ^9^ Østfold Hospital Trust Grålum Norway; ^10^ Institute of Medical Biology Faculty of Health Sciences University of Tromsø ‐ The Arctic University of Norway Tromsø Norway

**Keywords:** athymic nude fox1^nu^ mice, brain metastasis model, human melanoma, site specificity, tissue‐specific metastasis

## Abstract

**Background:**

Malignant melanomas frequently metastasize to the brain, but metastases in the cerebellum are underrepresented compared with metastases in the cerebrum.

**Methods:**

We established animal models by injecting intracardially in athymic nude fox1^nu^ mice two human melanoma cell lines, originating from a cerebral metastasis (HM19) and a cerebellar metastasis (HM86).

**Results:**

Using magnetic resonance imaging (MRI), metastases were first detected after a mean of 34.5 days. Mean survival time was 59.6 days for the mice in the HM86 group and significantly shorter (43.7 days) for HM19‐injected animals (*p* < 0.001). In the HM86 group, the first detectable metastasis was located in the cerebellum in 15/55 (29%) mice compared with none in the HM19 group (*p* < 0.001). At sacrifice, cerebellar metastases were found in 34/55 (63%) HM86‐injected mice compared with 1/53 (2%) in the HM19‐injected (*p* < 0.001) mice. At that time, all mice in both groups had detectable metastases in the cerebrum. Comparing macroscopic and histologic appearances of the brain metastases with their clinical counterparts, the cell line‐based tumors had kept their original morphologic characteristics.

**Conclusions:**

The present work demonstrates that human brain‐metastatic melanoma cells injected intracardially in mice had retained inherent characteristics also in reproducing interaction with subtle microenvironmental brain tissue compartment‐specific features. The models offer new possibilities for investigating tumor‐ and host‐associated factors involved in determining tissue specificity of brain metastasis.

## Graphical abstract

We present two new models that offer novel possibilities for investigating tumor‐ and host‐associated factors involved in malignant melanoma brain metastasis in general and tissue‐specific metastasis in particular. Two cell lines derived from a cerebral and a cerebellar human‐brain metastasis gave rise to two completely different patterns of spread, where 62.9% of animals injected with the cell line derived from a cerebellar human metastasis developed cerebellar metastases compared with 2% in the group injected with the cerebral cell line.

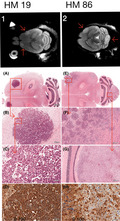

## 1 INTRODUCTION

The human skin is the largest organ in the body and its melanocytes offer protection against radiation from the sun.^1^, ^2^, ^3^ Regretfully, this protection is incomplete and sun exposure is the number one primary cause of cutaneous malignant melanoma (MM) in Caucasians.^4^, ^5^ Worldwide, the incidence of MM has increased in Caucasian populations over the last 15 years^6^, ^7^, ^8^, ^9^, ^10^ and it will likely increase further if preventive efforts are not undertaken.^11^ In Norway, the incidence has increased nearly 15‐fold since the Norwegian Cancer Registry was established in 1952, and in the age group 15–54 years, MM is now the second most common cancer type for both sexes. In 2015, the age‐adjusted incidence rate for men and women was 39.5 and 35.5 per 100,000 person‐years, respectively,^12^ making Norway one of the countries with the highest incidence of this malignancy.^13^

Brain metastases can occur with most cancer types.^14^ In clinical series, 80% of the brain metastases are found in the cerebrum and 20% in the posterior fossa (cerebellum and brain stem).^15^ This distribution closely parallels the regional blood flows and blood volumes as well as the relative brain tissue mass of the respective regions.^16^, ^17^ Cutaneous MM frequently metastasize to the brain^22^ in contrast to uveal melanomas.^23^ Interestingly, Rogne et al.^14^ found that brain metastases from MMs were significantly underrepresented in the cerebellum, accounting for only 5% of all the intracranial MM metastases resected in their neurosurgical series, and not 15%–20% as might be expected. This reduced propensity of MM to establish growth in the cerebellum is likely associated with microenvironmental and tumor cell‐associated factors,^14^, ^24^ in accordance with the seed and soil theory of metastasis. However, it is difficult to study associations between molecular and immunologic tumor characteristics and the occurrence and biologic behavior of brain metastasis in clinical series.^18^ Preclinical studies of brain metastases have been limited by the scarcity of clinically relevant models.^19^ We have previously reported several brain metastasis models in immunodeficient mice and rats^20^, ^21^; however, none of these are suited for studying tissue‐specific growth within the brain. We have established two in vitro cell lines directly from human MM brain metastases, of which one originates from a cerebellar and one from a cerebral metastasis in the respective patients. Melanoma brain metastasis animal models were developed by injecting the cell lines intracardially in female athymic nude fox1^nu^ mice. Our first objective was to examine whether the pattern of spread observed in the clinic could be reproduced in mice. The appearance and growth of resulting metastases in the animals were followed by MRI, examining patterns of metastasis to the cerebrum and cerebellum. The second objective was to study the relationship between tumor phenotype and metastatic site by morphologic and immunohistochemical analyses, comparing the mouse brain metastases to each other and to the clinical metastases from which the cell lines originated.

## 2 METHODS

### 2.1 Human tissue collection and mutation analyses

Fresh biopsy material was obtained during surgery at the Department of Neurosurgery, Oslo University Hospital. The tumor tissue was collected in 50 ml tubes filled with RPMI 1640 (Merck KgaA, Sigma‐Aldrich, catalog number R0883) and transported to the Department of Tumor Biology, Institute for Cancer Research, Oslo University Hospital. All patients had signed a written consent before tumor material was collected and the study was approved by The Regional Ethical Committee, Region South‐East, Norway (approval number 2011/2183). Immediately upon arrival in the laboratory, the tissue was split into samples for subsequent molecular analyses, which were stored at −70°C, samples for evaluation of tumor cell content, and one sample for the establishment of cell lines. DNA and RNA were isolated using the AllPrep DNA/RNA/miRNA Universal Kit (Qiagen GmbH, catalog number 80224), and the QiaCube system. Targeted DNA sequencing was conducted using the Ion AmpliSeq Cancer Hotspot Panel (v2) (LifeTechnologies, catalog number 4475346) covering ~2800 hotspot mutations in 50 cancer‐related genes. Data from the Ion PGM sequencing platform runs were processed by The Torrent Suite Variant Caller using panel customized parameters. Additional cutoff filters (e.g. minimum allele frequency) and manual evaluation by visual inspection were applied. In the patient brain metastases from where the two cell lines were established, the following gene aberrations were detected: HM19–BRAF K601E, TP53 S241F, APC R1105R, VHL P138S, HM86–NRAS Q61K.

### 2.2 Cell culture

Cell lines from two different patients were developed; HM19 originated from an MM metastasis in the cerebrum, whereas HM86 was derived from an MM metastasis in the cerebellum. Approximately one‐third of the tissue received was chopped into small pieces with a scalpel in a petri dish. The small tissue pieces were resuspended in the transport medium (RPMI 1640) and filtered through a 70 µm Falcon filter (Corning Incorporated, catalog number 431751) into a new 50 ml tube. The petri dish was washed with 2–5 ml RPMI 1640 supplemented with 10% FBS (Sigma‐Aldrich, catalog number F7524). The filter was also washed with this solution and all collected in the 50 ml tube. After centrifugation at 235 *g* for 5 min, the pellet was resuspended in a 5 ml cell culture medium and placed in a 25 cm^2^ cell culture flask (Nunc, Thermo Fisher Scientific, catalog number 156367). The cells were cultivated in RPMI supplemented with 10% FBS and 2 mM stable Glutamin (Merck KgaA, Sigma‐Aldrich, catalog number G8541) at 37°C with 5% CO_2_. The medium was changed once a week. After some weeks in culture, and when appropriate confluence was achieved, the cells were detached by 0.01 M EDTA (Merck KgaA, Sigma‐Aldrich, catalog number E8008) and split into new flasks. After 10 passages, we consider a cell line to be established. Both cell lines were routinely tested for Mycoplasma (VenorGem Mycoplasma detection kit, Minerva Biolabs GmbH, catalog number 11‐1100), while the human origin and identity were confirmed by genomic fingerprinting using the PowerPlex 16 kit (Promega, catalog number DC6530). For LV injections in mice, the cells were detached by EDTA, washed once, and resuspended in RPMI without FBS to a concentration of 5 × 10^5^ cells/0.2 ml.

### 2.3 Animals

In our nude rodent facility at the Department for Comparative Medicine, we bred female athymic nude fox1^nu^ mice (*N* = 120, aged >4 weeks). No more than 10 animals were placed in each cage. To minimize infection, the cages were kept in a pathogen‐free environment at constant temperature (21.5 ± 0.5°C) and humidity (55 ± 5%), 15 air changes/h, and a 12 h light/dark cycle. All procedures and experiments involving animals were approved by The National Animal Research Authority (FOTS ID: 7310) and carried out according to the European Convention for the Protection of Vertebrates used for Scientific Purposes. Food and water were daily controlled.

### 2.4 Left ventricular injection of the heart

The mice were anesthetized with a subcutaneous injection of Zoletil^®^ 5 µg/g body weight (Virbac Laboratories, catalog number A5727) and placed in a supine position on a styrofoam board. Parasternal on the left side, an incision was made to localize the third intercostal space where the punction site is. The tumor cells were introduced to the left ventricle of the heart by a 27‐gauge cannula connected to a 10 cm plastic tube (Valu‐set, BD Bioscience, Franklin Lakes, catalog number 387412). This arrangement was mounted on a stereotactic instrument to increase the accuracy. Because of the increased pressure in the left ventricle, we could assess if the punction was successful by observing oxygenated blood in the plastic tube. Tumor cells (5 × 10^5^) in 200 µl were injected. After the injection, we sealed the skin with one absorbable suture (Polysorb 5‐0; Covidien, catalog number GL‐890) and Histoacryl^®^ (B. Braun, Rubi, Spain, catalog number 1050052), after observing pulsating blood in the plastic tube. After the 10 min procedure, the animals were placed in cages often.

### 2.5 Magnetic resonance imaging

MRI was carried out at the MRI Core Facility at Department for Comparative Medicine, Oslo University Hospital, by using a Bruker Biospec 7 Tesla small‐animal scanner. An anatomically shaped receive‐only coil array (2 × 2) was placed over the head of the animal (Bruker BioSpin), and an 86 mm 1H volume resonator was used as a transmit coil. Mice were sedated with 5% Sevofluran^®^ (Baxter, catalog number 460‐220‐12) in O_2_ and maintained under general anesthesia (3% Sevofluran^®^). Respiration rate and body core temperature were monitored throughout the procedure by using an abdominal pressure‐sensitive probe and a rectal temperature probe (Small Animal Instruments).

Coronal and axial *T*_2_‐weighted images were obtained by using a fast spin‐echo sequence (RARE) with a repetition time (TR) of 2500 ms, an echo time (TE) of 35 ms, a RARE factor of 8, an image matrix of 256 × 256, a field of view (FOV) of 2 × 2 cm^2^, a slice thickness of 0.5 mm, a slice gap of 0.3 mm, and averages of 2. Scan time was 2:40 min.

MRI was used to locate the sites of metastatic intracranial lesions and to monitor the tumor growth. An initial baseline MRI scan was performed 24 h after injection of tumor cells to evaluate normal brain anatomy. The animals were then scanned once weekly for 5 weeks, and thereafter every tenth day until sacrifice.

The following additional parameters were recorded: location of the first intracranial lesion, time from intracardiac injection until first intracranial metastasis, locations and number of subsequent metastases, and time from injection until sacrifice.

The experimental endpoint was determined by the onset of neurologic symptoms, unacceptable weight loss (>15%) and body condition, or hind‐limb paralysis. At the endpoint, the mice were again scanned and subsequently sacrificed by cervical dislocation.

### 2.6 Animal tissue collection and preparation

After the experimental endpoint, the brain was surgically removed from the cranium. We made an incision in the level of C3 and dissected our way to the foramen magnum. From the foramen magnum, we performed a craniectomy from the medulla oblongata to the olfactory bulb. By stump dissection, the brain was free and placed on a sterile glass slide, where it was photographed and immediately placed in a solution of formalin and sent to the Department of Pathology, Oslo University Hospital for 3 days for sectioning and immunohistochemical analyses.

For the immunohistochemical staining, the Dako EnVision FLEX system (DakoCytomation, catalog number GV82311‐2) was used. Formalin‐fixed paraffin‐embedded sections (thickness 3 µm) from mouse brains were treated (97°C for 20 min and cooled down to 65°C) with the PT‐link and FLEX Target Retrieval Solution (High pH = 9.0) for deparaffinization, rehydration, and target retrieval (catalog number GV80411‐2). To block endogenous peroxidase, the sections were treated with EnVision Peroxidase‐Blocking Reagent (catalog number S202386‐2) for 5 min. The sections were incubated either with Melan‐A antibody (Abcam, catalog number GR111252‐2) dilution 1:250, S100 antibody (DakoCytomation, catalog number #27862) dilution 1:1500 or with anti‐HMB‐45 (DakoCytomation, catalog number #4684) dilution 1:75 for 30 min.

The sections were then incubated for 30 min with either labeled Polymer HRP antimouse (Dako) for HMB‐45 or labeled Polymer HRP antirabbit (Dako) for Melan‐A and S100. Then they were stained for 10 min with 3′3‐diaminobenzidine tetrahydrochloride (DAB), counterstained with hematoxylin, dehydrated, and mounted in Cytoseal XYL (Thermo Fisher Scientific, catalog number 8312‐4). The sections were deparaffinized for 10 min with xylol followed by rehydration for hematoxylin and eosin (HE) staining. Subsequently, the sections were stained for 6 min with hematoxylin and then cleaned with water before they were stained for 1 min with eosin. Diatex was used for dehydration and mounting the sections.

Sections were analyzed using a Mirax Scanner, Zeiss Microsystems GmbH. Scanning objective 20×, resolution 230 nm/pixel.

### 2.7 Data analysis

All images were analyzed using the open‐source DICOM viewer OsiriX (OsiriX version 7.0.2). Scans taken at the endpoint were examined retrospectively after sacrifice, allowing for easier identification of the seminal lesion. MRI scans were controlled by a senior neurosurgeon.

### 2.8 Statistics

Statistical analyses were performed using SPSS 21 (IBM). A sample size calculation was done in order to evaluate how many animals were needed in each group in order to get significant results. We performed a chi‐squared test with the following parameters; *α* = 0.05 and *β* = 0.2 (power = 1 − *β*) to calculate the needed sample size. According to the power analysis, 49 mice per cell line were needed. Allowing for 20% incidental loss of animals, the total sample size was 120 mice, resulting in 60 mice in each group for metastasis evaluation. Nine mice died after the injections and five mice failed to develop metastases in the HM 86 group, and seven in the HM 19 group, leaving 108 mice included in this study. We used the chi‐square test when comparing the distribution of metastases between the two groups. Survival and time to first detected metastases were measured using a Kaplan–Meier plot. A log‐rank test was used to evaluate the *p*‐value.

## 3 RESULTS

**FIGURE 1:**
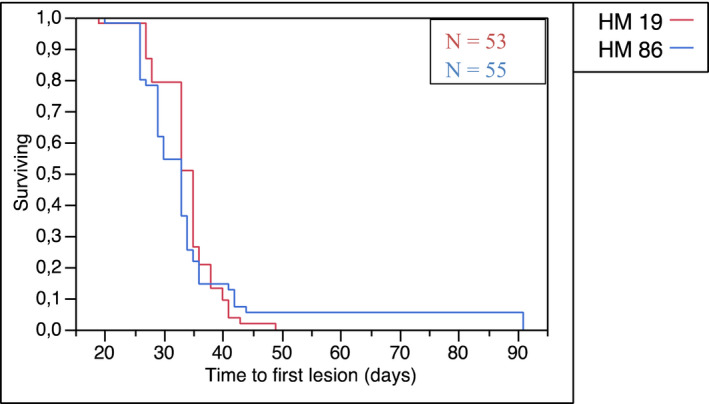
Kaplan–Meier curve. Time to first detected lesion (in days) for the two cell lines. No significant difference in time to detection of the first metastasis was found using a log‐rank test; *z* = 2.03, *p* = 0.423

One hundred and twenty female athymic nude fox1^nu^ mice were injected, of which nine died after intracardiac injections. Nine new animals were then injected, for a total number of 120. Twelve of 120 animals failed to develop any metastases (10%), leaving 108 mice for further analysis. The mean time from cell injection to first MRI‐detected intracranial metastasis was 34.5 days. By using a log‐rank test, we found no statistically significant difference between the two groups injected with the different cell lines (*z* = 2.03, *p* = 0.423) (Figure 1).

### 3.1 Metastasis location, development, clinical symptoms, and overall survival

**FIGURE 2:**
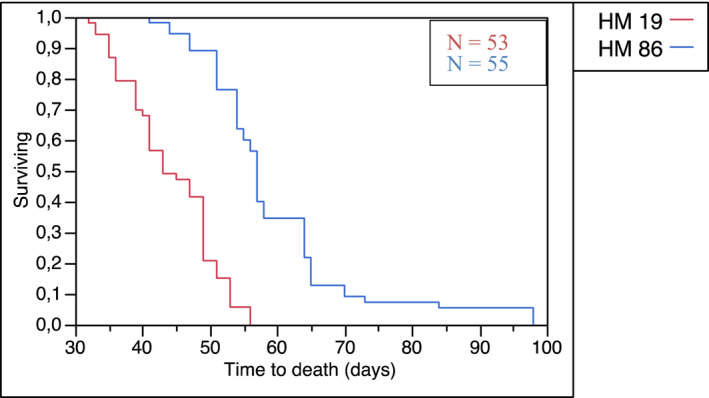
Kaplan–Meier curve. A significant difference between the two cell lines in overall survival was found using a log‐rank test; *z* = 4.84, *p* < 0.001

**FIGURE 3:**
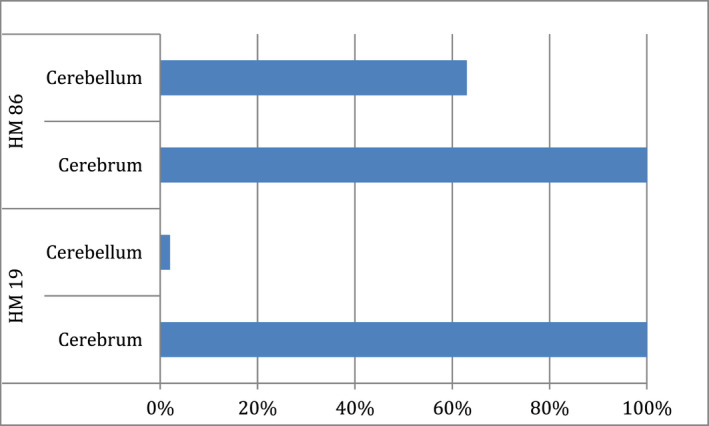
Cerebellar and cerebral lesions in mice injected with the two cell lines. On the *x*‐axis, the total percentage of mice with brain metastases at the time of sacrifice is shown. On the *y*‐axis, the two groups, namely HM 86 and HM 19, and the site of metastases (cerebellum and cerebrum) are presented. In the group injected with HM19, only 2% (1/53) of the animals developed a cerebellar metastasis compared with 62.9% (34/55) in the group injected with HM 86 cell. By using a chi‐square test, we found a significant relationship between the distribution of metastases in the cerebellum between the two cell lines. HM 86 are more likely than HM119 to metastasize to the cerebellum, Χ^2^ (1, *N* = 108) = 44.26, *p* < 0.01

For all 53 mice that received HM19 cells, the first detected intracranial metastasis was in the cerebrum. The lesions were well demarcated and widely distributed within the brain. Within weeks 4–8, the lesions did grow in both size and number. Animals in the HM19 group had a consistent and increasing development of symptoms with time. After an average of 43.8 days, the mice had either developed hind‐limb paralysis, respiratory failure, or a weight loss >15% and were sacrificed (Figure 2). At the time of sacrifice, only one mouse (2%) had developed metastasis in the cerebellum (Figure 3). Notably, 15 animals (30%) injected with HM19 cells eventually developed leptomeningeal lesions. Animals with these types of lesions typically expressed elevated respiratory frequency, cyanosis, and fatigue presumably due to respiratory failure secondary to brain stem compression.

Fifty‐five mice injected with HM86 cells were available for analysis. In this group, the first detected intracranial metastasis was in the cerebellum of 15 mice (29%). From week 4 to week 6, the lesions did increase slowly in size and number, but from week 7 onward, the lesions did grow rapidly and the animals in this group had a dramatic and rapid course of their illness. The mean overall survival time in this group was 59.6 days, significantly longer than with HM19 cells (log‐rank test: *z* = 4.84, *p* < 0.001) (Figure 2). At the time of sacrifice, as many as 34 animals (62.9%), had developed cerebellar metastases, but at that time, all mice also had lesions in the cerebrum (Figure 3).

Using the chi‐square test, we found a significant relationship between the distribution of metastases in the cerebellum between the two cell lines. HM 86 are more likely than HM119 to metastasize to the cerebellum, *X* ^2^ (1, *N* = 108) = 44.26, *p* <0.01.

### 3.2 Metastasis morphology and immunohistochemistry

**FIGURE 4:**
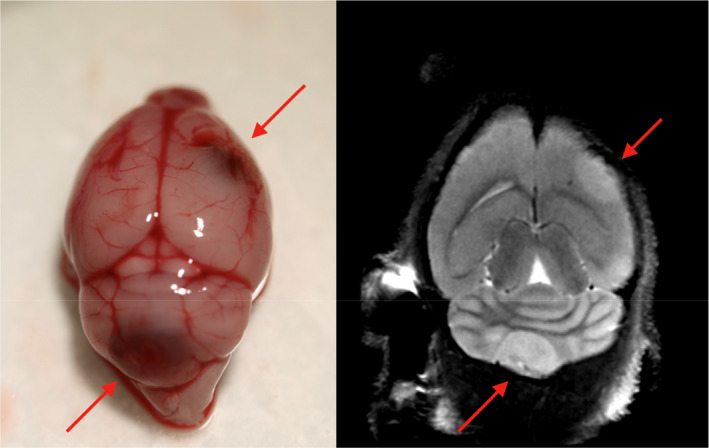
Left: Representative photo of the brain from a mouse injected with the cell line HM86. The red arrows point to one large metastasis central in the cerebellum and one cerebral metastasis in the right hemisphere. Right: the corresponding MRI scan was taken a few hours before the mice were sacrificed, confirming the metastases at the two locations

**FIGURE 5:**
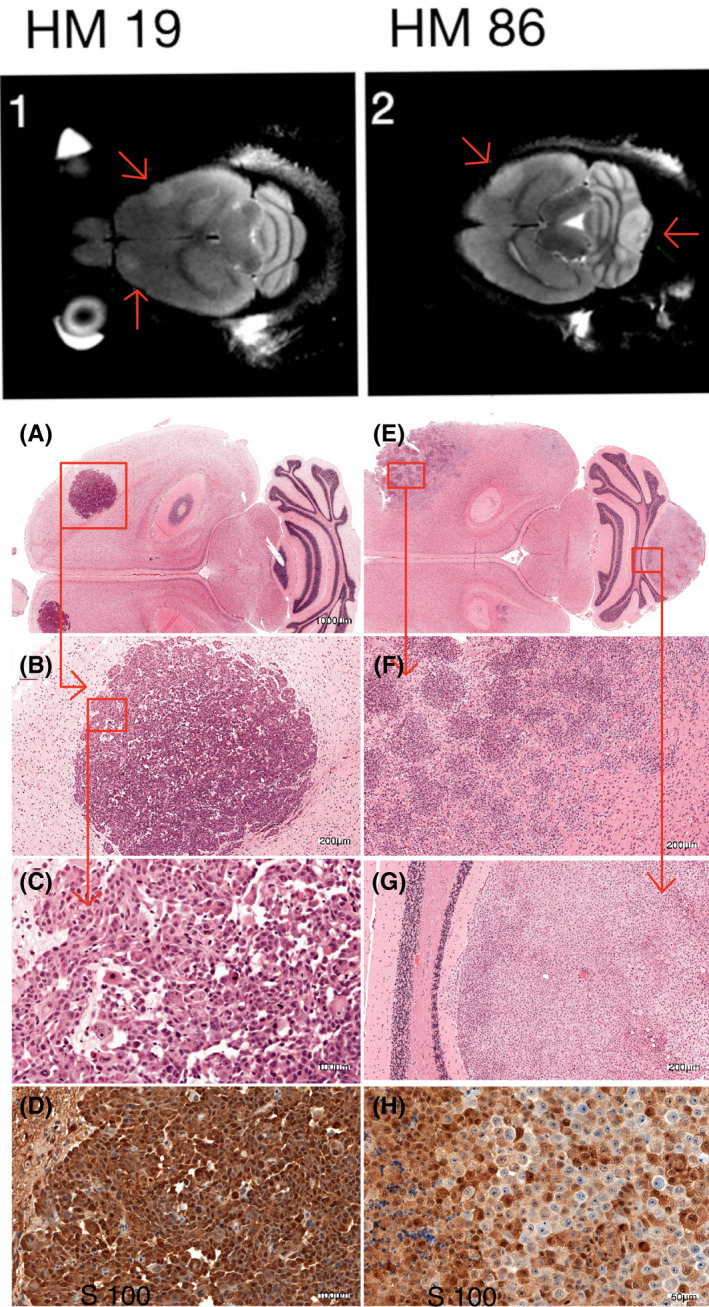
The left column represents mice injected with HM 19 and the right with HM 86. Figures 1 and 2 are *T*_2_‐weighted MRI scans. Arrows point to the metastases. Pictures A, B, and C show the cerebral metastasis in a mouse injected with HM19 cells at increasing magnification and standard H&E staining (scale bar: A: 1000 µm, B: 200 µm, C: 100 B 200 µm, C: 100 µm) and picture D shows an IHC analysis of the same metastasis stained for S100 (Scale bar 100µm). Picture E and F show a cerebral metastasis in a mouse injected with HM86 cells at increasing magnification (scale bar: E: 1000 µm, F 200 (scale bar E: 1000 µm, F: 200 µm). There is a distinct difference in growth pattern compared with the corresponding cerebellar metastasis (G) (scale bar: 200 µm). Picture H shows the same lesion as depicted in G, but at higher magnification (scale bar: 50 µm), and stained for S100

**FIGURE 6:**
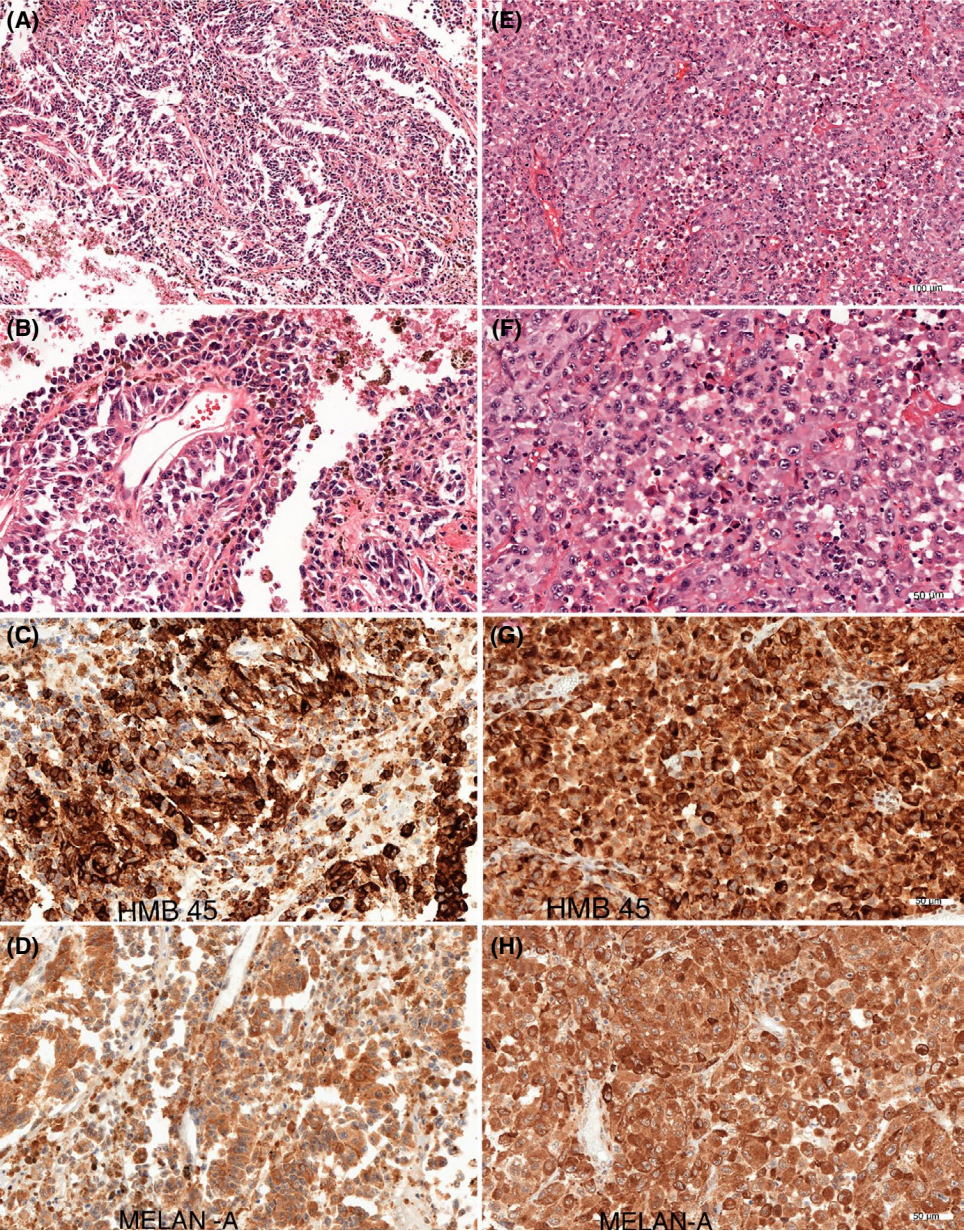
Histologic sections of the original patients’ brain metastases giving rise to the cell line HM 19 (left) and HM 86 (right). The magnification can be seen using the scale bar in the lower right corner (A and E = 100 µm, and B, C, D, F, G, H = 50 µm). Left column (A) H&E staining of the cerebral metastasis, HM 19. (B) The same metastasis but with greater magnification. (C) IHC analysis for HMB‐45. (D) IHC analysis for Melan‐A. Right column; (E) H&E staining of the cerebellar metastasis, HM 86. (F) the same metastasis but with greater magnification. (G) IHC analysis for HMB‐45. (H) IHC analysis for Melan A

Macroscopic examination of the mouse brains confirmed the location and size of the metastases detected by MRI, as exemplified in Figure 4. H&E sections from the macroscopic metastases in mice were taken in a plane similar to the corresponding MRIs to make them comparable to the imaged tumors (Figure 5). Tissue samples from the original brain metastases in the patients as well as brain metastases in mice were stained with melanoma markers Melan‐A, S100, and HMB‐45 and were all dominated by positive cells. Comparing the morphologic appearance in tumor sections (Figures 5 and 6), we found that the metastases arising from cell line HM19 had a nodular pattern with cells growing in a nest‐like pattern, with large necrotic areas and mitoses. These features were evident in samples both from the patient and mouse metastases. Metastases arising from the HM86 cell line differed from HM19 and showed a more epithelioid growth pattern as well as cells with spindle‐like features. The latter was most prominent in the cerebral lesions, whereas the cerebellar lesions showed a more typical epithelioid picture. These tumors also had fewer necrotic areas, but the same amount of mitoses. The HM86 cells were less pigmented than the HM19 cells in both the human material and in metastases in mice.

## 4 DISCUSSION

It is well known that different cancer types have different propensities for metastasizing to the brain, but most studies do not differentiate between the locations of the metastasis within the various brain compartments. Rogne et al.^14^ reported that in a large clinical series of patients with MMs, cerebellar metastases were significantly underrepresented compared with what is reported for other cancer types. If blood flow or tissue volume was the decisive factor, MM metastases should occur in the cerebrum in 80% of the cases, brain stem in 5%, and cerebellum in 15%. However, Rogne et al.^14^ found that the rate of cerebellar MM metastases was as low as 5%. To identify underlying causes of this difference, one has to search among the multitude of factors that may affect the metastatic process to the brain.^25^

**FIGURE 7:**
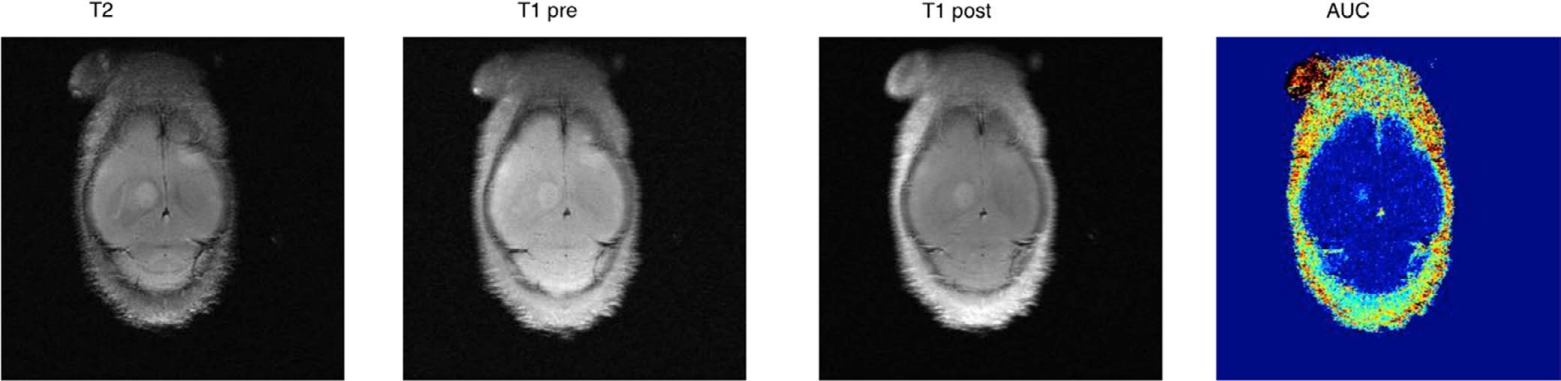
Starting from left to right are four MRI images of the same mouse, but with different modalities. The first is a regular *T*_2_‐weighted scan showing a clearly visible metastasis central in the cerebrum as well as a frontal lesion in the right hemisphere. The next image is a *T*_1_‐weighted scan where the lesions still are visible, but less clear. The third picture is a *T*_1_‐weighted scan using gadolinium as a contrast agent. The fourth picture is a semi‐quantitative descriptive method that measures the area under the contrast curve (AUC). Studies in test animals showed that the use of a contrast agent was redundant

We wanted to develop models that allow the study of this biologically interesting phenomenon. For this purpose, we used intraventricular heart injections in athymic nude fox1^nu^ mice of two melanoma cell lines originating from a cerebral metastasis (HM19) and a cerebellar metastasis (HM86). We postulated that the HM19 cell line would follow a similar pattern in an experimental brain metastasis model in immune‐deficient mice, whereas the HM86 cell line would show a higher propensity to metastasize to the cerebellum. We initially used contrast‐enhanced MRI (CE‐MRI), but the metastases were better visualized in a regular *T*_2_‐weighted sequence (Figure 7). Our observation corresponds with those of several studies that describe a low sensitivity of CE‐MRI in animal models because of an intact BBB early in the metastasis development, or where the tumor grows to utilize preexisting capillaries.^26^, ^27^, ^28^, ^29^ Serial *T*_2_W MRI proved excellent to detect and follow the development of intracranial spread in the mice, and two distinct patterns of metastasis for the two MM cell lines were observed, reflecting their origins.

We found a significant overrepresentation of cerebellar metastases in mice injected with the HM86 cell line compared with those injected with HM19 cells using a chi‐square test (*X* ^2^ (1, *N* = 108) = 44.26, *p* < 0.01). This finding is remarkable and warrants further studies of the underlying factors involved. Notably, the metastases in the mice were also morphologically similar to their respective original human brain metastases. The cerebellar tumors were more nodular and less vascularized as well as well demarcated compared with the cerebral ones. Classical melanoma markers like Melan‐A and S100 were positive in both cell line tumors and in the tumors of origin.

**FIGURE 8:**
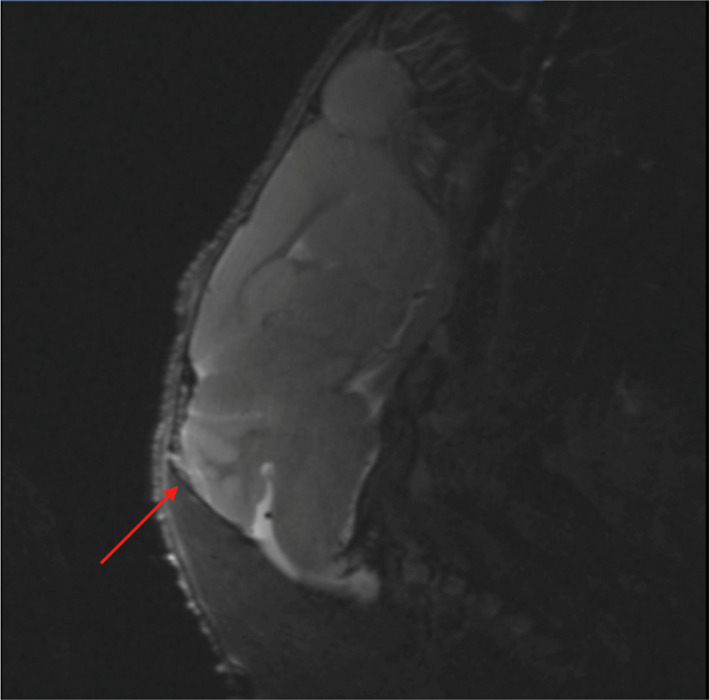
*T*_2_‐weighted MRI, sagittal view of leptomeningeal metastasis in the meninges over the cerebellum

Interestingly, as many as 28% of the HM19 mice had leptomeningeal metastases at the time of sacrifice (Figure 8). Such metastases may become manifest first at a late stage of the disease, possibly not being easily observed in cases with very aggressive disease. However, it is known that clinical leptomeningeal MM metastases are associated with rapid disease progression with a mean survival of only 8–10 weeks after being diagnosed.^30^ The HM19 cell line may provide a missing research tool also for studying the biology of this disease manifestation.^17^, ^19^, ^30^, ^31^

The intracardiac injection of cells results in hematogenous spread throughout the animals, in our case with a very good take rate (90%). However, this approach also has its clear limitations as 500,000 tumor cells were injected over a very short period into the systemic circulation, a situation that can lead to intravascular cancer cell aggregates, and misguided injections may result in perfusion to the pulmonary circulation or thorax. In both cases, this can lead to premature death, which occurred in nine of our mice (7%). Furthermore, the wide distribution of cancer cells may give rise to metastases anywhere in the body, which might lead to death before brain metastases are detectable. An alternative approach to obtain experimental brain metastases is to inject the cells directly into the internal carotid artery.^20^, ^32^ This provides a high tumor load to the brain but may be less suitable to study homing mechanisms. Intracarotid injection also demands microsurgical skills, is more time‐consuming, and possesses a higher risk of mortality or stroke. In addition to the two methods mentioned above, intrathecal injections give meningeal tumors,^33^, ^34^ and last, cells may also be injected orthotopically into the brain.^35^ Despite the limitation associated with the intracardiac injection model, the approach gives several advantages compared with other available options for studying brain metastases, a notion supported by the present results. Thus, only one of 53 mice injected with HM19 cells, originating from a cerebral metastasis, developed cerebellar metastases in the mice, compared with 34 of 54 animals injected with HM86 cells from a cerebellar metastasis. That all HM86‐injected mice also had cerebral manifestations at the end of their somewhat long life span may reflect that over time absolute tissue specificity is very rare.

Microenvironmental factors are essential for metastatic growth and include among other release of soluble factors and bidirectional interaction between cancer cells and the microenvironment.^36^, ^37^, ^38^ Such factors may affect tumor cell growth, motility, invasion, and angiogenesis^39^, ^40^, ^41^ and might be mediated, for example, by expression of cell surface molecules encoding receptors or ligands,^42^ microRNAs,^43^ exosomes,^44^ and metabolic reprogramming.^45^, ^46^ The preference of HM86 cells for growth in the cerebellum is likely the result of a complex interplay between tumor cells and microenvironmental factors. Such factors may also explain the small differences in morphology between HM86 metastases in the cerebellum and cerebrum.

In summary, the present work demonstrates that human brain‐metastatic melanoma cells, even after being cultured in vitro for a long period, have retained important characteristics in their interaction with subtle microenvironmental brain tissue‐associated differences in mice. The two new models offer novel possibilities for investigating tumor‐ and host‐associated factors involved in MM brain metastasis in general and in determining tissue‐specific metastasis in particular. This is in line with the comments in a recent review paper in which the authors emphasize the need for new animal models for studying brain metastasis.^18^

## ETHICS STATEMENT

All patients have signed a written consent form before tumor material was collected and the study was approved by the Regional Ethical Committee, Region South‐East, Norway (approval number 2011/2183). All procedures and experiments involving animals were approved by the National Animal Research Authority (FOTS ID: 7310) and carried out according to the European Convention for the Protection of Vertebrates used for Scientific Purposes.

## CONFLICT OF INTEREST

No conflict of interest.

## Data Availability

The data used to support the findings of this study are included in the article. Raw data are available from the corresponding author, upon reasonable request.

## References

[1] Baroni A , Buommino E , De Gregorio V , Ruocco E , Ruocco V , Wolf R . Structure and function of the epidermis related to barrier properties. Clin Dermatol. 2012;30(3):257‐262. 22507037 10.1016/j.clindermatol.2011.08.007

[2] Lee SH , Jeong SK , Ahn SK . An update of the defensive barrier function of skin. Yonsei Med J. 2006;47(3):293‐306. 16807977 10.3349/ymj.2006.47.3.293 PMC2688147

[3] Singer AJ , Clark RA . Cutaneous wound healing. N Engl J Med. 1999;341(10):738‐746. 10471461 10.1056/NEJM199909023411006

[4] Wagner JD , Gordon MS , Chuang TY , Coleman JJ 3rd . Current therapy of cutaneous melanoma. Plast Reconstr Surg. 2000;105(5):1774‐1799. 10809113 10.1097/00006534-200004050-00028

[5] Sober AJ , Chuang T‐Y , Duvic M , et al. Guidelines of care for primary cutaneous melanoma. J Am Acad Dermatol. 2001;45(4):579‐586. 11568750 10.1067/mjd.2001.117044

[6] Erdmann F , Lortet‐Tieulent J , Schüz J , et al. International trends in the incidence of malignant melanoma 1953–2008—are recent generations at higher or lower risk? Int J Cancer. 2013;132(2):385‐400. 22532371 10.1002/ijc.27616

[7] Garbe C , Leiter U . Melanoma epidemiology and trends. Clin Dermatol. 2009;27(1):3‐9. 19095149 10.1016/j.clindermatol.2008.09.001

[8] Giblin AV , Thomas JM . Incidence, mortality and survival in cutaneous melanoma. J Plast Reconstr Aesthet Surg. 2007;60(1):32‐40. 17126264 10.1016/j.bjps.2006.05.008

[9] Godar DE . Worldwide increasing incidences of cutaneous malignant melanoma. J Skin Cancer. 2011;2011:1‐6. 10.1155/2011/858425 PMC3191827 22007306

[10] Jemal A , Saraiya M , Patel P , et al. Recent trends in cutaneous melanoma incidence and death rates in the United States, 1992‐2006. J Am Acad Dermatol. 2011;65(5):S17.e1‐S17.e11. 22018063 10.1016/j.jaad.2011.04.032

[11] Guy GP Jr , Thomas CC , Thompson T , et al. Vital signs: melanoma incidence and mortality trends and projections—United States, 1982–2030. MMWR Morb Mortal Wkly Rep. 2015;64(21):591‐596. 26042651 PMC4584771

[12] Norway CRo . Cancer in Norway 2016—Cancer incidence, mortality, survival and prevalence in Norway. 2016.

[13] Geisler J , Bachmann IM , Nyakas M , et al. Malignant melanoma–diagnosis, treatment and follow‐up in Norway. Tidsskr nor Laegeforen. 2013;133(20):2154‐2159. 24172628 10.4045/tidsskr.12.1416

[14] Rogne SG , Helseth E , Brandal P , Scheie D , Meling TR . Are melanomas averse to cerebellum? Cerebellar metastases in a surgical series. Acta Neurol Scand. 2014;130(1):1‐10. 24313862 10.1111/ane.12206

[15] Delattre JY , Krol G , Thaler HT , Posner JB . Distribution of brain metastases. Arch Neurol. 1988;45(7):741‐744. 3390029 10.1001/archneur.1988.00520310047016

[16] Zarrinkoob L , Ambarki K , Wahlin A , Birgander R , Eklund A , Malm J . Blood flow distribution in cerebral arteries. J Cereb Blood Flow Metab. 2015;35(4):648‐654. 25564234 10.1038/jcbfm.2014.241 PMC4420884

[17] Gavrilovic IT , Posner JB . Brain metastases: epidemiology and pathophysiology. J Neurooncol. 2005;75(1):5‐14. 16215811 10.1007/s11060-004-8093-6

[18] Eroglu Z , Holmen SL , Chen Q , et al. Melanoma central nervous system metastases: an update to approaches, challenges, and opportunities. Pigment Cell Melanoma Res. 2019;32(3):458‐469. 30712316 10.1111/pcmr.12771 PMC7771318

[19] Cohen JV , Tawbi H , Margolin KA , et al. Melanoma central nervous system metastases: current approaches, challenges, and opportunities. Pigment Cell Melanoma Res. 2016;29(6):627‐642. 27615400 10.1111/pcmr.12538 PMC5398760

[20] Myklebust AT , Helseth A , Breistol K , Hall WA , Fodstad O . Nude rat models for human tumor metastasis to CNS. Procedures for intracarotid delivery of cancer cells and drugs. J Neurooncol. 1994;21(3):215‐224. 7699416 10.1007/BF01063770

[21] Rye PD , Norum L , Olsen DR , Garman‐Vik S , Kaul S , Fodstad O . Brain metastasis model in athymic nude mice using a novel MUC1‐secreting human breast‐cancer cell line, MA11. Int J Cancer. 1996;68(5):682‐687. 8938153 10.1002/(SICI)1097-0215(19961127)68:5<682::AID-IJC20>3.0.CO;2-2

[22] Barnholtz‐Sloan JS , Sloan AE , Davis FG , Vigneau FD , Lai P , Sawaya RE . Incidence proportions of brain metastases in patients diagnosed (1973 to 2001) in the Metropolitan Detroit Cancer Surveillance System. J Clin Oncol. 2004;22(14):2865‐2872. 15254054 10.1200/JCO.2004.12.149

[23] Lorigan JG , Wallace S , Mavligit GM . The prevalence and location of metastases from ocular melanoma: imaging study in 110 patients. AJR Am J Roentgenol. 1991;157(6):1279‐1281. 1950883 10.2214/ajr.157.6.1950883

[24] Paget S . The distribution of secondary growths in cancer of the breast. 1889. Cancer Metastasis Rev. 1989;8(2):98‐101. 2673568

[25] Pienta KJ , Robertson BA , Coffey DS , Taichman RS . The cancer diaspora: Metastasis beyond the seed and soil hypothesis. Clin Cancer Res. 2013;19(21):5849‐5855. 24100626 10.1158/1078-0432.CCR-13-2158 PMC3835696

[26] Zhou H , Chen M , Zhao D . Longitudinal MRI evaluation of intracranial development and vascular characteristics of breast cancer brain metastases in a mouse model. PLoS ONE. 2013;8(4):e62238. 23638013 10.1371/journal.pone.0062238 PMC3639286

[27] Leenders W , Küsters B , Pikkemaat J , et al. Vascular endothelial growth factor‐A determines detectability of experimental melanoma brain metastasis in GD‐DTPA‐enhanced MRI. Int J Cancer. 2003;105(4):437‐443. 12712432 10.1002/ijc.11102

[28] Zhang RD , Price JE , Fujimaki T , Bucana CD , Fidler IJ . Differential permeability of the blood‐brain barrier in experimental brain metastases produced by human neoplasms implanted into nude mice. Am J Pathol. 1992;141(5):1115‐1124. 1443046 PMC1886664

[29] Lockman PR , Mittapalli RK , Taskar KS , et al. Heterogeneous blood‐tumor barrier permeability determines drug efficacy in experimental brain metastases of breast cancer. Clin Cancer Res. 2010;16(23):5664‐5678. 20829328 10.1158/1078-0432.CCR-10-1564 PMC2999649

[30] Smalley KS , Fedorenko IV , Kenchappa RS , Sahebjam S , Forsyth PA . Managing leptomeningeal melanoma metastases in the era of immune and targeted therapy. Int J Cancer. 2016;139(6):1195‐1201. 27084046 10.1002/ijc.30147 PMC4939138

[31] Padilla‐Vazquez F , Escobar‐de la Garma VH , Ayala‐Arcipreste A , Mendizabal‐Guerra R , Cuesta‐Mejia T . Melanocytoma and meningeal melanocytosis, similar but different lesions. Cir Cir. 2017;85(3):273‐278. 28126183 10.1016/j.circir.2016.11.006

[32] Schackert G , Price JE , Zhang RD , Bucana CD , Itoh K , Fidler IJ . Regional growth of different human melanomas as metastases in the brain of nude mice. Am J Pathol. 1990;136(1):95‐102. 2297053 PMC1877453

[33] Myklebust AT , Godal A , Fodstad O . Targeted therapy with immunotoxins in a nude rat model for leptomeningeal growth of human small cell lung cancer. Cancer Res. 1994;54(8):2146‐2150. 8174121

[34] Kooistra KL , Rodriguez M , Powis G , et al. Development of experimental models for meningeal neoplasia using intrathecal injection of 9L gliosarcoma and Walker 256 carcinosarcoma in the rat. Cancer Res. 1986;46(1):317‐323. 3753551

[35] Daphu I , Sundstrøm T , Horn S , et al. In vivo animal models for studying brain metastasis: value and limitations. Clin Exp Metastasis. 2013;30(5):695‐710. 23322381 10.1007/s10585-013-9566-9

[36] De Wever O , Mareel M . Role of tissue stroma in cancer cell invasion. J Pathol. 2003;200(4):429‐447. 12845611 10.1002/path.1398

[37] Schmidt‐Hansen B , Klingelhöfer J , Grum‐Schwensen B , et al. Functional significance of metastasis‐inducing S100A4(Mts1) in tumor‐stroma interplay. J Biol Chem. 2004;279(23):24498‐24504. 15047714 10.1074/jbc.M400441200

[38] Cooper CR , Chay CH , Gendernalik JD , et al. Stromal factors involved in prostate carcinoma metastasis to bone. Cancer. 2003;97(3 Suppl):739‐747. 12548571 10.1002/cncr.11181

[39] Nygaard V , Prasmickaite L , Vasiliauskaite K , Clancy T , Hovig E . Melanoma brain colonization involves the emergence of a brain‐adaptive phenotype. Oncoscience. 2014;1(1):82‐94. 25593989 10.18632/oncoscience.11 PMC4295759

[40] Kulesa PM , Kasemeier‐Kulesa JC , Teddy JM , et al. Reprogramming metastatic melanoma cells to assume a neural crest cell‐like phenotype in an embryonic microenvironment. Proc Natl Acad Sci U S A. 2006;103(10):3752‐3757. 16505384 10.1073/pnas.0506977103 PMC1450149

[41] Eichhoff OM , Zipser MC , Xu M , et al. The immunohistochemistry of invasive and proliferative phenotype switching in melanoma: a case report. Melanoma Res. 2010;20(4):349‐355. 20526217 10.1097/CMR.0b013e32833bd89e PMC2901773

[42] Hamidi H , Ivaska J . Author correction: every step of the way: integrins in cancer progression and metastasis. Nat Rev Cancer. 2019;19(3):179. 30705430 10.1038/s41568-019-0112-1

[43] Hanniford D , Zhong J , Koetz L , et al. A miRNA‐based signature detected in primary melanoma tissue predicts development of brain metastasis. Clin Cancer Res. 2015;21(21):4903‐4912. 26089374 10.1158/1078-0432.CCR-14-2566 PMC4631639

[44] Hoshino A , Costa‐Silva B , Shen T‐L , et al. Tumour exosome integrins determine organotropic metastasis. Nature. 2015;527(7578):329‐335. 26524530 10.1038/nature15756 PMC4788391

[45] Bettum IJ , Gorad SS , Barkovskaya A , et al. Metabolic reprogramming supports the invasive phenotype in malignant melanoma. Cancer Lett. 2015;366(1):71‐83. 26095603 10.1016/j.canlet.2015.06.006

[46] Lim S‐O , Li C‐W , Xia W , et al. EGFR signaling enhances aerobic glycolysis in triple‐negative breast cancer cells to promote tumor growth and immune escape. Cancer Res. 2016;76(5):1284‐1296. 26759242 10.1158/0008-5472.CAN-15-2478 PMC4775355

